# Navigating Uncertain Waters: First-Trimester Screening’s Role in Identifying Neonatal Complications

**DOI:** 10.3390/jcm13071982

**Published:** 2024-03-29

**Authors:** Grzegorz Swiercz, Anna Zmelonek-Znamirowska, Karol Szwabowicz, Justyna Armanska, Karolina Detka, Marta Mlodawska, Jakub Mlodawski

**Affiliations:** 1 Collegium Medicum, Jan Kochanowski University in Kielce, Zeromskiego Street 5, 25-369 Kielce, Poland; 2 Clinic of Obstetrics and Gynecology, Provincial Combied Hospital in Kielce, Grunwaldzka 45, 25-736 Kielce, Poland

**Keywords:** first-trimester screening, neonatal outcome, pregnancy complications

## Abstract

**Background**: Contemporary diagnostic methods aimed at assessing neonatal outcomes predominantly rely on the medical history of pregnant women. Ideally, universal biomarkers indicating an increased risk of delivering infants in poor clinical condition, with a heightened likelihood of requiring hospitalization in a Neonatal Intensive Care Unit (NICU), would be beneficial for appropriately stratifying pregnant women into a high-risk category. Our study evaluated whether biochemical and ultrasonographical markers universally used in first-trimester screenings for non-heritable chromosomal aberrations could serve this purpose. **Methods**: This study encompassed 1164 patients who underwent first-trimester screening, including patient history, ultrasound examinations, and biochemical tests for pregnancy-associated plasma protein-A (PAPP-A) and the free beta-HCG subunit (fbHCG), from January 2019 to December 2021. The research concentrated on the correlation between these prenatal test results and neonatal outcomes, particularly Apgar scores, umbilical blood pH levels, and the necessity for NICU admission. **Results**: In our cohort, neonates scoring lower than 8 on the Apgar scale at birth exhibited lower concentrations of PAPP-A in the first trimester, both in raw and normalized values (PAPP-A MoM 0.93 vs. 1.027, *p* = 0.032). We also observed a higher pulsatility index in the venous duct in the first trimester in full-term neonates born with <8 points on the Apgar scale. Additionally, newborns born with an umbilical blood pH < 7.2 had lower normalized first-trimester PAPP-A concentrations (0.69 vs. 1.01 MoM, *p* = 0.04). We also noted that neonates requiring NICU hospitalization post-delivery had lower first-trimester bHCG concentrations (0.93 MoM vs. 1.11 MoM, *p* = 0.03). However, none of the correlations in our study translated into a robust prognostic ability for predicting dichotomous outcomes. All areas under the curve achieved a value < 0.7. **Conclusions**: Low concentrations of PAPP-A and free bHCG subunit in the first trimester may be associated with poorer clinical and biochemical conditions in neonates post-delivery. However, the relationship is weak and has limited predictive capability. Further research evaluating these relationships is necessary for the appropriate stratification of pregnant women into high-risk categories for neonatological complications.

## 1. Introduction

Complications in newborns associated with poor birth conditions sometimes occur in pregnancies classified as low risk. Unexpected complications in newborns happen in up to 29% of pregnancies where no perinatal risk factors were identified [1]. Research indicates that some perinatal complications, such as meconium-stained amniotic fluid, forceps delivery, and vacuum delivery, may occur more frequently in low-risk than in high-risk pregnancies [1]. Therefore, identifying risk factors that can better stratify pregnant women into appropriate groups is of the utmost importance.

In cases where newborns are born in poor clinical condition from pregnancies where no prior risk factors for such an event were diagnosed, several potential problems may arise. The child might be born in a facility not equipped to handle neonatal complications, lacking the necessary equipment, knowledge, and experience to quickly secure the newborn’s health. Such a newborn might require transfer to another center, which entails additional risk for complications and requires extra involvement of specialized staff and equipment [2]. This is particularly important in countries with large areas and low population density where centers are significantly distant from each other. For children of pregnant women in the high-risk group for complications such as NICU hospitalization, in utero transportation to the appropriate center during the onset of labor could be beneficial for both mother and child. It is well-known that many perinatal complications cannot currently be predicted, and our knowledge is based only on the existence of risk factors. However, searching for risk factors is vital from the viewpoint of the current trend in research methodology; risk factors can later be used as variables in regression equations and machine learning.

Optimal stratification of patients into a high-risk group for perinatal complications should occur in the first trimester of pregnancy. This could allow for the escalation of care for pregnant women to a higher level of referral from the outset. First-trimester prenatal screenings are part of a screening program that should be offered to every pregnant woman in most countries, regardless of her baseline risk of non-heritable chromosomal aberrations [3]. This screening is based on a medical interview, ultrasonographic examination with quantitative marker measurements, qualitative marker assessments, and biochemical testing for pregnancy-associated plasma protein-A (PAPP-A) and free beta-HCG subunit (fbHCG). Biochemical test results, before being included in the calculation, are normalized in relation to gestational age and other variables such as ethnic origin, smoking, conception method, weight, age, etc., according to the norms developed by the Fetal Medicine Foundation. (PAPP-A MoM and fbHCG MoM) [4]. Besides calculating the risk of chromosomal aberrations, markers can also indicate other complications related to the fetus, such as fetal growth restriction (FGR), preterm labor (PTL), and risk for preeclampsia (PE) [5].

There are limited studies directly translating the results of biochemical and ultrasonographic tests in mothers to neonatal outcomes. The state of the newborn is one of the most clinically significant outcomes, as perinatal complications can evolve into long-term problems. These persistent issues can imply the need for specialized care, rehabilitation, mental and physical burden for parents and caregivers, financial strain, and resource consumption [6]. In our analysis, we decided to examine how first-trimester biochemical tests and the quantitative parameter in the form of the Pulsatility Index (PI) in the ductus venosus (DV), measured in the first trimester of pregnancy, impact neonatal outcomes after birth, measured clinically and biochemically.

## 2. Materials and Methods

Patients who underwent first-trimester screening tests at the Prenatal Diagnostic Clinic of the Provincial Integrated Hospital in Kielce’s Department of Obstetrics and Gynecology from January 2019 to December 2021 and subsequently gave birth at the same clinic were selected for the study. During the first-trimester screening, patient interviews were conducted using a standard form necessary for calculating the risk of chromosomal aberrations according to the Fetal Medicine Foundation (FMF) guidelines. Ultrasound scans were then performed in adherence to FMF guidelines [7], measuring the crown-rump length (CRL), assessing the nuchal translucency (NT) and nasal bone (NB), examining the flow in the ductus venosus (DV) with pulsatility index (PI) evaluation, as well as checking the fetal heart rate (FHR) and conducting scans for anatomical abnormalities. Patients with detected fetal anomalies in the first trimester were excluded from the study. Additionally, patients who were lost to follow-up before delivery (including those who experienced a miscarriage before the 23rd week of pregnancy) were also excluded. All examinations were conducted by physicians holding current FMF certificates for prenatal screenings and certificates from the Polish Society of Gynecologists and Obstetricians for prenatal examinations, with at least three years of experience in first-trimester prenatal diagnostics. Blood samples were collected on the same day for analysis to determine the levels of PAPP-A and fbHCG. All biochemical measurements were conducted between the 11th and 14th weeks of pregnancy. The assays were performed using a BRAHMS Kryptor analyzer, with variable normalization accomplished using FMF 2012 software (version 3.0), based on the gestational age calculated from the crown-rump length (CRL). We obtained approval from the bioethics commission at Jan Kochanowski University in Kielce for patient research (approval number—51/2023). All methods were carried out in compliance with the relevant guidelines and regulations of the ethical commission. All participants provided informed consent for ultrasonographic diagnosis and participation in the study.

We conducted a comprehensive follow-up on all patients, gathering data on the clinical and biochemical status of newborns post-delivery. The clinical assessments included evaluating the Apgar score within the first minute of life for identifying newborns in moderate to severe distress ([Apgar score < 8]) and those in severe distress ([Apgar score < 4]), as well as reassessments at the 10 min mark. The Apgar scores were assigned by a neonatologist present at the delivery. The analysis of clinical outcomes was conducted for both the entire cohort and specifically for full-term newborns, to isolate the effect of prematurity on Apgar scores. Other variables of interest were the incidence of low umbilical blood pH levels (<7.2 and <7.1), which were predetermined as indicators of potential future abnormal neurological development in the child [8].

Immediately following the clamping of the umbilical cord, blood was collected from the umbilical vein for gas analysis using the ABL800FLEX analyzer. We also evaluated the necessity for neonatal intensive care unit (NICU) admission and instances of a low Ponderal index (defined as a value < 2, with the Ponderal index being calculated via the formula: mass/height^3). Comparisons between groups were made based on the differences in the medians of the respective parameters. For outcomes where differences were observed, we generated receiver operating characteristic (ROC) curves and calculated the area under the curve (AUC) along with a 95% confidence interval.

For continuous variables, the median was used to describe the central tendency, and the interquartile range (IQR) was used to describe dispersion. The Mann–Whitney U test was employed for comparing variables. A *p*-value of <0.05 was considered indicative of statistical significance. Statistical analyses were conducted using Statistica 13.1 (TIBCO Software Inc., Palo Alto, CA, USA) and IBM SPSS Statistics version 26.

## 3. Results

We included 1164 patients in the study. The demographic characteristics of the group are presented in Table 1. The percentage of complications used as outcomes in our analysis is shown in Table 2. We treated all outcomes as grouping variables (excluding cases where no complication was noted in the groups) and compared the median concentrations of fb-HCG, fb-HCG MoM, PAPP-A, PAPP-A MoM, and the Pulsatility Index (PI) in the ductus venosus (DV) (Table 3). We observed that full-term newborns born with an Apgar score of less than 8 in the first and tenth minutes of life had a statistically significantly higher PI in the DV in the first trimester. For the general population, it was noted that newborns born with an Apgar score of <8 in the first minute of life had significantly lower concentrations of PAPP-A and PAPP-A MoM, as well as higher DV PI in the first trimester. We also observed that newborns born with umbilical cord blood pH < 7.2 had lower first-trimester PAPP-A MoM concentrations. PAPP-A and PAPP-A MoM were also lower in the group of newborns born with a Ponderal index < 2. We found that newborns requiring post-birth hospitalization in the neonatal intensive care unit (NICU) had lower first-trimester concentrations of fb-HCG and fb-HCG MoM. For all continuous variables with median differences in groups defined by a particular outcome discriminator, we plotted receiver operating characteristic (ROC) curves. In Table 4, we present the area under curve (AUC) values for each predictor. For DV in predicting a reduced Apgar score < 8 points at 1 min, no significant difference from 0.5 AUC was found. For other predictors, a significant AUC difference from 0.5 was observed (*p* < 0.05). The highest AUC was for predicting umbilical cord blood pH < 7.2 (AUC = 0.669, 95% CI = 0.508–0.829) by PAPP-A MoM. However, all obtained values demonstrated poor or failed prognostic ability with AUC values < 0.7 [9].

## 4. Discussion

Our analysis reveals several significant correlations between variables assessed during first-trimester screening and neonatal outcomes. However, the literature regarding these correlations is not consistent. In a 2022 study conducted in a Turkish center, first trimester biochemical test results were retrospectively analyzed in patients who had low-risk pregnancies between 37 and 41 weeks, and whose children required NICU hospitalization. The control group comprised patients whose newborns had uncomplicated postnatal outcomes. Mothers of newborns hospitalized in the NICU had lower first-trimester PAPP-A levels (0.91 vs. 1.12 PAPP-A MoM, *p* < 0.001). The study did not show a statistically significant difference in fbHCG levels (median 1.05 ± 0.46 for NICU newborns vs. 1.11 ± 0.49 for the control group, *p* = 0.134). However, a lower fbHCG MoM median value was noted in children born with an Apgar score < 7 in the first minute of life (0.89 ± 0.23 vs. 1.1 ± 0.49, *p* < 0.05), confirming a correlation between poorer clinical status and lower biochemical parameters in the first trimester [10].

In a Danish study conducted among women who underwent prenatal testing between January 2005 and December 2007, 15.2% of newborns (up to 4 weeks old) from a population of 9450 patients were admitted to the NICU. The study showed significantly lower PAPP-A MoM levels in pregnancies where the newborn was admitted to the NICU compared to the control group. PAPP-A levels < 0.4 MoM were associated with a higher chance of NICU hospitalization (adjusted odds ratio (aOR) = 1.6, 95% CI = 1.2–2.0), regardless of concurrent prematurity and a lower birth weight. Moreover, levels below this cutoff extended NICU stay compared to newborns with PAPP-A MoM > 0.4. Other adverse outcomes associated with low PAPP-A levels included neonatal hypoglycemia (aOR = 3.2, 95% CI = 1.7–6.1), neonatal jaundice (OR = 2.02, 95% CI = 1.35–3.03), and an Apgar score < 7 at five minutes (OR = 4.1, 95% CI = 1.4–12.1) [11]. Our study similarly found lower PAPP-A levels in newborns born in moderate to severe conditions in the first minute of life. However, the difference was not significant when only full-term newborns were considered. Like our results, the cited study demonstrated a correlation between first-trimester fbHCG levels and the risk of NICU hospitalization. fbHCG was significantly lower in newborns admitted to the NICU, a difference significant for both the entire population and full-term newborns born between 39 and 40 weeks. Newborns whose mothers had first trimester fbHCG < 0.4 MoM had a 1.5 times greater chance of NICU hospitalization (aOR = 1.5; 95% CI = 1.1–2.1), but unlike PAPP-A, fbHCG did not correlate with the length of NICU stay. Low fbHCG levels also correlated with an increased risk of neonatal jaundice, but no correlation was found with neonatal hypoglycemia or an Apgar score < 7 at 5 min. In another study, Sirikunalai et al. [12] demonstrated that low first-trimester fbHCG levels were associated with a greater risk of a low Apgar score (lower than 7 at the 5th minute) (relative ratio (RR) = 3.11, 95% CI = 2.05–4.71). The other literature indicates that fbHCG levels below the 5th percentile are associated with a threefold greater chance of pregnancy loss before 24 weeks and a twofold greater risk of loss after 24 weeks [13]. In our cohort, miscarriage was an exclusion criterion.

Reduced PAPP-A levels appear to be an independent risk factor for Respiratory Distress Syndrome (RDS) and the need for surfactant administration in newborns. For PAPP-A levels less than 1 MoM, the chance of RDS in a newborn was more than eight times higher (OR: 8.2, 95% CI = 1.2–55.6) [14].

An interesting finding from our cohort analysis was the correlation between first trimester ductus venosus (DV) pulsation and an Apgar score of less than 8 in the first minute of life. The literature shows a relationship between high DV pulsation and adverse fetal outcomes in fetuses without structural abnormalities or chromosomal aberrations. The prevalence of fetal loss during pregnancy for a fetus with a first-trimester DV Pulsatility Index (PI) greater than the 95th percentile was 4.4%, compared to 0.3% in the rest of the cohort in the Indian population [15]. In the Turkish population, it was observed that patients with a DV PI greater than 1.22 had a higher risk of stillbirth, miscarriage, Fetal Growth Restriction (FGR), and major congenital heart defects. However, no correlation with lower Apgar scores was observed in this population [16]. We found no other studies confirming such a dependency. In our cohort, this correlation might be due to the exclusion of all fetuses with chromosomal aberrations and severe congenital anomalies, which could have been indications for pregnancy termination. However, we did not exclude patients who might give birth to children with mild heart defects, for which abnormal flow in the DV is undoubtedly an early ultrasonographic marker [17].

Lower biochemical marker levels may be linked to poorer neonatal outcomes and are often associated with various pregnancy complications, including preterm labor (PTL). This link persists even in full-term pregnancies. The relationship between neonatal outcomes and marker levels is subject to debate within the scientific community, which may stem from variations in the prevalence of pregnancy complications across populations, differing research methodologies, the timing of biochemical marker testing, and the types of bioassays employed. Additionally, challenges in study comparisons could result from varying regional guidelines on the use of acetylsalicylic acid for the prevention of Fetal Growth Restriction (FGR) and Preeclampsia (PE) across medical centers. In our study, the Polish recommendations for acetylsalicylic acid usage were to include cases with a PE risk higher than 1:150, as assessed in the first trimester, and PAPP-A levels under 0.4 MoM [8]. Notably, some referenced studies were conducted before the ASPRE study’s findings, which significantly altered the management of high-risk pregnancies with low PAPP-A levels for PE and FGR [18]. Despite these variations, a consensus in the literature suggests that lower levels of biochemical markers correlate with an increased risk of neonatal complications. Conversely, elevated levels of fbHCG and PAPP-A have a negligible effect on adverse pregnancy outcomes [19].

Certainly, the relationships we have examined do not indicate a causal link. Changes in biochemical marker concentrations should be considered as risk factors, rather than definitive indicators of adverse obstetric events. We understand that aside from chromosomal aberrations, the variation in biochemical marker concentrations also correlates with the risk of fetal growth restriction, pre-eclampsia, and iatrogenic preterm birth [20]. Furthermore, our study was conducted in a period following the ASPRE trial [18], which altered the approach to preventing some of these complications. Nevertheless, our findings suggest that despite the potential for preventative measures, a low concentration of PAPP-A remains a risk factor for adverse obstetric events.

The limitation of our study is its retrospective nature. We considered the general population over the entire time period, but did not include the use of acetylsalicylic acid by pregnant women as a differentiating factor. We also did not consider many confounding factors that could affect the final neonatal outcome. It should also be remembered that despite its common use in studies, the Apgar score remains a somewhat subjective marker of a newborn’s state. The advantage of our study is the use of biochemical assessment of the newborn in the form of umbilical cord blood pH evaluation.

## 5. Conclusions

Low concentrations of PAPP-A and βhCG, along with an elevated pulsation in the ductus venosus, may be associated with adverse neonatal birth conditions, a decreased Ponderal index, and an elevated likelihood of neonatal intensive care unit (NICU) admission. These observed relationships necessitate further multicenter studies.

## Figures and Tables

**Table 1:** Demographic characteristics of the group.

Parameter	Value
Height [mean]	165 cm (SD = 13.33)
Weight [mean]	66 kg (SD = 7.88)
BMI [mean]	24.82 kg/m^2^ (SD = 4.13)
IVF pregnancy	4.3% (n = 50)
1st pregnancy	37.8% (n = 440)
Pre-pregnancy diabetes	1.12% (n = 13)
Pre-pregnancy hypertension	0.6% (n = 7)
High risk for trisomy 21 (>1:300)	7.98% (n = 93)
Intermediate risk for trisomy 21 (1:301–1:1000)	14% (n = 163)
High risk for trisomy 18 (>1:300)	0.68% (n = 8)
Intermediate risk for trisomy 18 (1:301–1:1000)	1.97% (n = 1164)
High risk for trisomy 13 (>1:300)	0.51% (n = 6)
Intermediate risk for trisomy 13 (1:301–1:1000)	0.77% (n = 9)
PAPP-A < 0.4 MoM	3.78% (n = 44)

**Table 2:** Prevalence of complications occurring as neonatal outcomes in the study.

Newborn Outcome	Prevalence
Apgar < 8 1st minute	6.18% (72)
Apgar < 4 1st minute	0.94% (11)
Apgar < 8 10th minute	1.11% (13)
Apgar < 4 10th minute	0% (0)
Apgar < 8 1st minute term pregnancy	4.46% (52)
Apgar < 4 1st minute term pregnancy	0.06% (7)
Apgar < 8 10th minute term pregnancy	0.52% (6)
Apgar < 4 10th minute term pregnancy	0% (0)
intensive care unit hospitalization	5.58% (n = 65)
low Ponderal index (<2)	25.25% (294)
pH < 7.2	1.03% (12/739)
neonatal death	0.3% (n = 4)
pH < 7.1	0%

**Table 3:** Median values of individual parameters in groups identified based on discriminators [*—*p* < 0.05].

	Term Apgar < 8 at 1st Minute				Term Apgar < 8 at 10th Minute		
	yes	no	*p*		yes	no	
fb-HCG	40.495	40.045	0.711	fb-HCG	43.245	40.025	0.977
fb-HCG MoM	1.244	1.112	0.263	fb-HCG MoM	1.265	1.114	0.491
PAPP-A	3.835	3.960	0.256	PAPP-A	4.380	3.950	0.975
PAPP-A MoM	0.944	1.038	0.118	PAPP-A MoM	1.111	1.032	0.742
DV PI	1.040	1.000	0.045 *	DV PI	1.110	1.010	0.254
	Term Apgar < 4 at 1st minute				General Apgar < 8 at 1st minute		
	yes	no			yes	no	*p*
fb-HCG	41.040	40.030	0.825	fb-HCG	39.920	39.515	0.509
fb-HCG MoM	1.066	1.117	0.633	fb-HCG MoM	1.278	1.102	0.128
PAPP-A	4.100	3.950	0.513	PAPP-A	3.500	3.870	0.040 *
PAPP-A MoM	0.932	1.033	0.966	PAPP-A MoM	0.939	1.027	0.032 *
DV PI	0.980	1.010	0.843	DV PI	1.070	1.000	0.001 *
	General Apgar < 8 at 10th minute				General Apgar < 4 at 1st minute		
	yes	no			yes	no	
fb-HCG	45.350	39.460	0.478	fb-HCG	39.570	33.260	0.635
fb-HCG MoM	1.326	1.104	0.348	fb-HCG MoM	1.110	1.066	0.411
PAPP-A	2.380	3.860	0.075	PAPP-A	3.860	2.380	0.104
PAPP-A MoM	0.720	1.023	0.217	PAPP-A MoM	1.023	0.720	0.251
DV PI	1.100	1.000	0.111	DV PI	1.005	1.010	0.276
	pH < 7.2				NICU admission		
	yes	no			yes	no	
fb-HCG	44.665	39.450	0.641	fb-HCG	28.830	40.025	0.002 *
fb-HCG MoM	1.127	1.090	0.564	fb-HCG MoM	0.930	1.118	0.034 *
PAPP-A	2.890	3.900	0.084	PAPP-A	3.540	3.885	0.053
PAPP-A MoM	0.694	1.018	0.045 *	PAPP-A MoM	0.925	1.027	0.128
DV PI	1.045	1.000	0.743	DV PI	1.010	1.000	0.176
	Neonatal death				Low Ponderal index (<2)		
	yes	no			yes	no	
fb-HCG	33.500	39.600	0.720	fb-HCG	38.475	39.685	0.713
fb-HCG MoM	0.936	1.110	0.647	fb-HCG MoM	1.112	1.104	0.564
PAPP-A	2.880	3.865	0.176	PAPP-A	3.595	3.940	0.031 *
PAPP-A MoM	0.895	1.018	0.791	PAPP-A MoM	0.934	1.056	0.001 *
DV PI	1.105	1.000	0.383	DV PI	1.005	1.010	0.682

**Table 4:** Values of AUC (area under curve) for individual continuous predictors in the prediction of adverse obstetric events, along with 95% confidence intervals [SE—standard error].

Outcome		AUC	SE	AUC Low 95%	AUC High 95%	*p*
Term Apgar < 8 at 1st minute	DV PI	0.583	0.043	0.499	0.668	0.053
General Apgar < 8 at 1st munute	PAPP-A	0.572	0.033	0.507	0.637	0.029
	PAPP-A MoM	0.575	0.034	0.508	0.642	0.028
	DV PI	0.621	0.035	0.552	0.689	0.001
pH < 7.2	PAPP-A MoM	0.669	0.082	0.508	0.829	0.039
Low Ponderal index	PAPP-A	0.542	0.019	0.504	0.580	0.031
	PAPP-A MoM	0.563	0.019	0.525	0.601	0.001
NICU hospitalization	fb-HCG	0.616	0.037	0.544	0.688	0.002
	fb-HCG MoM	0.578	0.039	0.501	0.655	0.048

## Data Availability

The dataset used for this study was uploaded to a public repository and is available at this URL: https://doi.org/10.17605/OSF.IO/XZ8RQ (accessed on 22 March 2024).
